# Hepatoid Adenocarcinoma of the Lung: A Systematic Review of the Literature From 1981 to 2020

**DOI:** 10.3389/fonc.2021.702216

**Published:** 2021-08-06

**Authors:** Zan Hou, Jiaqing Xie, Li Zhang, Gangyi Dai, Yuanhang Chen, Lang He

**Affiliations:** ^1^Department of Oncology, The Affiliated Fifth People’s Hospital of Chengdu University of TCM, Chengdu Fifth People’s Hospital, Chengdu, China; ^2^Department of Radiation Oncology, State Key Laboratory of Oncology in South China, Collaborative Innovation Center for Cancer Medicine, Sun Yat-Sen University Cancer Center, Guangzhou, China; ^3^Department of Clinical Laboratory, The Affiliated Fifth People’s Hospital of Chengdu University of TCM, Chengdu Fifth People’s Hospital, Chengdu, China; ^4^Department of Pathology, The Affiliated Fifth People’s Hospital of Chengdu University of TCM, Chengdu Fifth People’s Hospital, Chengdu, China

**Keywords:** hepatoid adenocarcinoma of the lung, clinical features, treatment, prognosis, case report, systematic review

## Abstract

**Objectives:**

We report the first case of hepatoid adenocarcinoma of the lung (HAL) with PIK3CA mutation. In addition, we analyzed data from HAL cases over the past 40 years to study its main treatment methods, prognosis, and the relationship between prognosis and the serum alpha-fetoprotein (AFP) level before treatment.

**Methods:**

We report a 66-year-old male case who was diagnosed with locally advanced HAL with PIK3CA mutation and carried out a systematic literature search for HAL cases documented between 1981 and 2020. General patient information including case characteristics was extracted and summarized. The median OS (mOS) of HAL patients was determined using the KM survival curve. The Cox proportional hazards regression model was used to evaluate the effect of tumor size, location, and serum AFP value before treatment and radical surgery (RS) on the prognosis of patients.

**Results:**

A total of 46 studies including 51 HAL patients was included in our review. Our study revealed that 52.9% of tumors were located in the upper lobe of the right lung. The proportion of serum AFP-positive patients before treatment, early-stage patients (TNM stage I and II), and patients who had received surgery were 69.2%, 34.1%, and 40%, respectively. The mOS of HAL patients was 16.0 months. The 2-year and 5-year survival rates of the patients were 35.3% and 8.0%, respectively. In the subgroup analysis, the 2-year survival rate for patients who received RS was 62.5%, while for patients who were unable to undergo RS, it was only 12.5% (*p* = 0.009). The Cox proportional hazards regression model indicated that RS can significantly improve the prognosis of HAL patients (*p* = 0.011), although the location and size of tumor as well as the serum AFP value before treatment had no significant effect on their prognosis (*p* = 0.82, *p* = 0.96, *p* = 0.25).

**Conclusions:**

HAL patients have a poor prognosis, and the survival benefits for patients receiving chemoradiotherapy or chemotherapy alone appear to be limited. We demonstrate statistically for the first time that pretreatment serum AFP values are not related to the prognosis of HAL patients and RS can significantly improve patient prognosis.

## Introduction

Hepatoid adenocarcinoma of the lung (HAL) is an extremely rare type of primary lung adenocarcinoma that shares similarities with hepatocellular carcinoma (1). HAL has been shown to produce some products of normal hepatocytes or hepatocellular carcinoma, such as ferritin and alpha-fetoprotein (AFP) (2). However, with the exception of its low incidence and poor prognosis, very little about is known about HAL. The first case of HAL was reported in 1981 by Yasunami et al. (3), while Grossman et al. (4) comprehensively summarized the clinical characteristics of HAL patients documented before 2016. Although this study described the clinical characteristics of 28 HAL patients, the overall survival (OS) data and factors affecting patient prognosis were not examined. More recently, Tonyali et al. (5) carried out a literature review of HAL in 2020, but this study only reviewed the clinical characteristics of 21 patients with HAL and therefore did not include all the documented cases.

By December 2020, although an increasing number of HAL cases had been reported, a summary analysis of all cases reported over the past 40 years had not been carried out. To date, the epidemiology, molecular pathology, effective treatment methods, and factors affecting the prognosis of HAL patients remain unclear. Therefore, in addition to reporting a HAL case with PIK3CA mutation, we have summarized and analyzed all the HAL cases reported in the literature between 1981 and 2020.

## Materials and Methods

### Study Design

The relationship between serum AFP value and prognosis of HAL patients remains unclear. Previous studies have suggested that the serum AFP value before treatment is related to the prognosis of HAL patients, with an initial high AFP level associated with a shorter OS time (6, 7). Since these findings are consistent with our case study, we conducted a systematic review of the literature in order to clarify the relationship between these two factors. We searched multiple databases for papers containing HAL patients that had been published in English between 1981 and 2020. Appropriate papers were screened out through strict inclusion and exclusion criteria. Finally, we were able to determine whether the serum AFP level before treatment significantly affected the prognosis of HAL patients by extracting useful data and conducting appropriate statistical analyses. We also briefly describe the epidemiology and overall prognosis of these patients. The study design is outlined below.

### Search Strategy

This study was conducted on the basis of the preferred reporting items for systematic reviews and meta-analysis (PRISMA) statement (8). A comprehensive literature search was conducted on papers published between 1981 and December 31, 2020. The Ovid MEDLINE, Ovid Embase, and Science Citation Index (Web of Science) were searched by two independent reviewers (Zan Hou and Jiaqing Xie) for eligible studies. The overall search strategy was (1) hepatoid (All Fields), (2) adenocarcinoma (All Fields), and (3) lung (All Fields). Searches in electronic databases combined the terms 1, 2, and 3.

### Study Selection Criteria

The eligibility of the studies was assessed by two independent reviewers (Zan Hou and Jiaqing Xie) who read titles, abstracts, and full texts. After a systematic screening, the following cases were excluded: (i) the case was not pathologically confirmed as HAL, (ii) the case was not diagnosed as primary HAL, and (iii) the literature did not contain the full text.

### Data Extraction

The following data were independently extracted from the included cases by two reviewers (Zan Hou and Jiaqing Xie): (1) Basic information such as year of publication, author’s name, patient’s gender, and age. (2) Case features including the location and size of the tumor, the serum AFP value before treatment, TNM staging, and gene status. (3) Treatment and prognosis data including the treatment method, whether adequate treatment was conducted, and prognosis. Inadequate treatment referred to patients who refused any or part of the treatment mentioned in the paper. OS was defined from the baseline to death from any cause or last follow-up.

### Statistical Analysis

General characteristics of all patients, including the average age, proportion of serum AFP-positive cases, and proportion of surgical treatment were summarized. The average value of continuous variables was expressed by mean ± standard deviation. KM survival curves were generated for all patients with survival data to evaluate the median OS (mOS) of all HAL patients with OS data. Survival rates were compared using the chi-square test. The size and location of tumor, serum AFP value before treatment, whether or not surgery was performed, and OS data of the patients were analyzed using the Cox proportional hazards regression model to determine whether these four indicators had a significant impact on the prognosis of the patients.

The OS data of patients who received inadequate treatment were excluded in any statistical analyses that included OS data. The size of tumor was expressed by its longest diameter. The serum AFP value of all patients with normal serum AFP before treatment was quantified as 7 ng/ml for statistical analysis. Radical surgery (RS) referred to lobectomy of primary lesion plus lymph node dissection. Cases were excluded for RS analysis that did not specify the surgical method, or when the patient underwent lobectomy alone or palliative surgical treatments.

## Results

**Figure 1:**
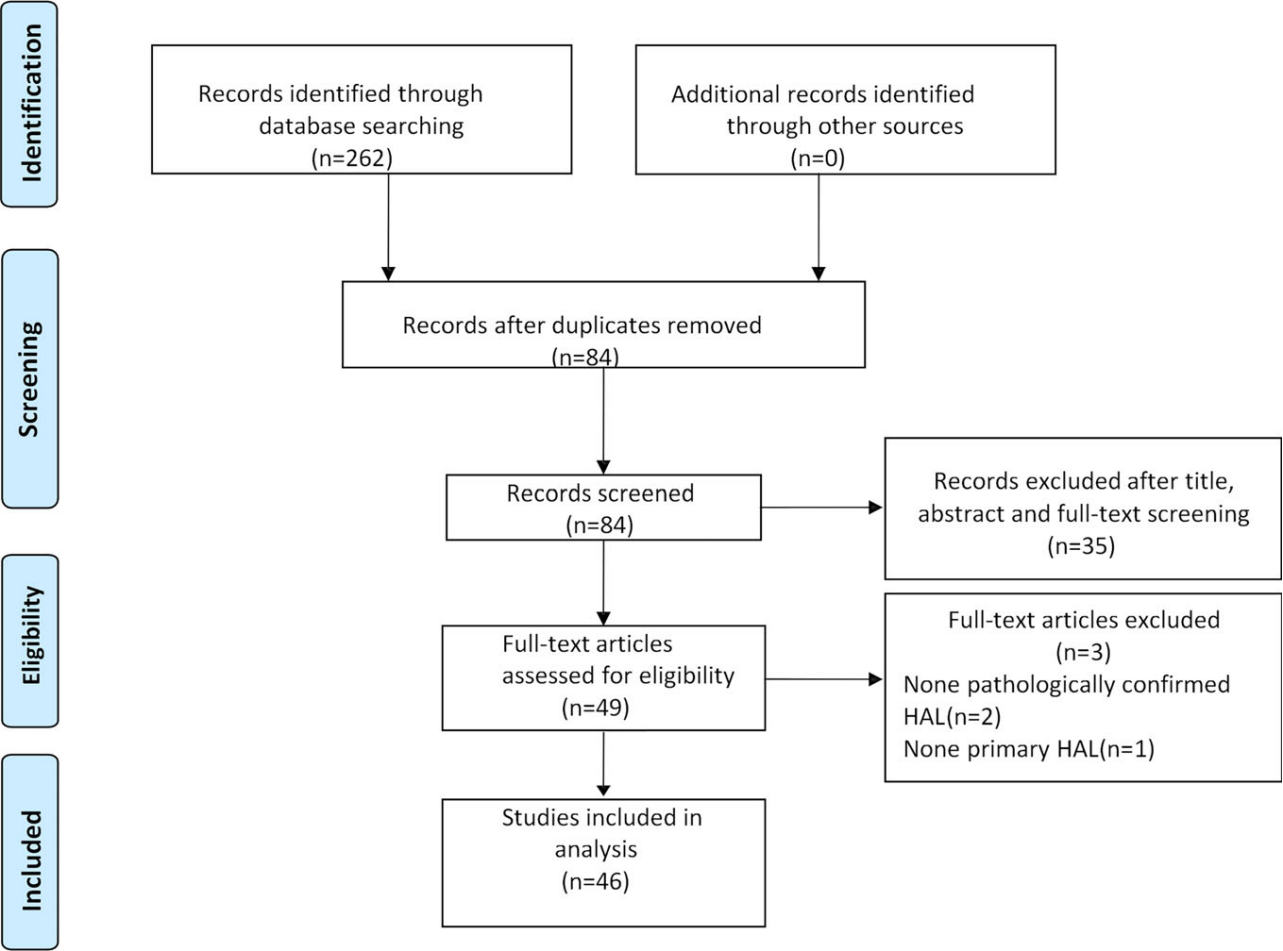
Paper selection flowchart.

After the selection procedure (**Figure 1**), 46 papers were considered eligible for our systematic review. The data of these cases are listed in **Table 1**. The total number of patients included in this study was 51.

**Table 1:** The clinical data of 51 cases.

Year	Author	Gender-Age (years)	Smoke	Location	Size (cm)	Serum AFP value before treatment (ng/ml)	TNM Stage	KI-67 and Gene mutation detection: Yes/No, Specific mutant gene	Treatment method	Complete treatment: Yes/No	Prognosis
1981	Yasunami (3)	Male-67	NM	Left upper lobe	8	19000	cT3N2Mx	NM	RT	Yes	16 months (dead)
1987	Miyake (9)	Male-73	NM	Left upper lobe	5×6×5	1039	NM	NM	RS+RT	Yes	18 months (dead)
1988	Saka (10)	Male-73	NM	Right upper lobe	3.9×3×3	289	pT2N0M0	NM	RS	Yes	28 months (alive)
1992	Okunaka (11)	Male-49	NM	Right upper lobe	6×5×5	9300	cT3NxMx	NM	RS	Yes	11 months (alive)
1997	Nasu (12)	Male-63	NM	Right upper lobe	14×13 ×12	14,000	cT4N2	NM	Chemo	Yes	11 months (dead)
1997	Arnould (13)	Male-36	Yes	Left upper lobe	11	6090	pT4N2	NM	Chemo	Yes	7 months (dead)
2000	Carlinfante (14)	Male-82	Yes	Left lower lobe	3.5	NM	cT2aN0M0	NM	Surgery (specific unknown)	Yes	84 months (alive)
2002	Hayashi (15)	Male-55	Yes	Right upper lobe	5×4.8×6.5	NM	pT2bN0M0	NM	RS	Yes	32 months (alive)
2002	Hiroshima (16)	Male-71	Yse	Right lower lobe	10.5×8.5×7	7417	pT3N1M0	NM	RS	Yes	12 months (dead)
2003	lino (17)	Male-63	NM	Right upper lobe	2.8×2.5	NM	pT1N0M0	NM	RS	Yes	5 months (alive)
2003	Terracciano (18)	Male-49	NM	Left lower lobe	5	203,320	pT2bNxM1	NM	Pulmonary lobectomy	Yes	2 months (dead)
2007	Ivan (19)	Male-54	Yes	Left upper lobe	13×11	14540	cT4N3M1	NM	Chemo+brain RT	Yes	NM
2007	Wu (20)	Male-50	Yes	Right upper lobe	6×5×5	Normal	cT2N1M0	NM	RS	Yes	45 months (alive)
2008	Kishimoto (21)	Male-64	NM	Left lower lobe	7.5×7×4	673	cT3N0M0	NM	Surgery (Specific unknown)	Yes	NM
2010	Fornasa (22)	Female-68	No	Left upper lobe	4.5×4×4	Normal	pT2b	NM	Chemo	Yes	15 months (alive)
2012	Khozin (23)	Female-56	Yes	Right anterior cardiophrenic angel; Right middle lobe	5.5; 1.8	Normal	cT4	YES,ALK +	Crizotinib	Yes	6months (dead)
2012	Valentino (24)	Male-71	No	Right lower lobe	1.8×1.5×1.5	1201	pT1N0M1	NM	①RS; ②Chemo+RT	Yes	8months (dead)
2012	Mokrim (25)	Male-52	Yes	Left upper lobe	11.8×12×8	5000	cT4N0M1	NM	Chemo	Yes	5months (dead)
2012	Papatsimpas (6)	Male-48	NM	Right upper lobe	20×11×8	39000	cT4	NM	①Chemo+bevacizumab; ②Erlotinib and palliative RT	Yes	6months (dead)
2013	Cavalcante (26)	Male-66	Yes	Right upper lobe	NM	Normal	cT4NxM1	NM	No treatment	NO	0.5month (dead)
2013	Lin (27)	Male-66	Yes	Right upper lobe	7.3×5.6× 3.3	8686	pT3N0M0	NM	RS	Yes	48 months (alive)
2014	Che (28)	Male-48	Yes	Left upper lobe	7.9×10	6283	pT4N1M0	NM	Chemoradiation	Yes	21 months (dead)
2014	Haninger (29)	Male-51	Yes	Right upper lobe	4.2×3.7	NM	cT2aN3M0	Ki-67 30%NM	Chemoradiation	Yes	14 months (dead)
		Male-52	Yes	Right upper lobe	2.5	NM	cT1bN0M1	Ki-67 10% NM	RS+ Chemoradiation	Yes	37 months (alive)
		Male-64	Yes	Left upper lobe	3.2×2.2	NM	cT2aN0M1	Ki-67 50%; Yes, None	Palliative surgery+ chemoradiation	Yes	10 months (dead)
		Female-54	Yes	Left upper lobe	1	NM	cT1aN0M1	Ki-67 10%	Chemoradiation	Yes	108 months (alive)
		Male-60	Yes	Right upper lobe	11.2×10.1×8.5	4410	cT3N2M1	Ki-67 10%	Chemoradiation	Yes	1 month (alive)
2014	Shaib (30)	Female-53	Yes	Right upper lobe	9.5×9.0×8.0	37,810	pT3N0M0	NM	RS+ Chemo	Yes	48 months (alive)
2015	Gavrancic (31)	Male-64		Right upper lobe	3.8×2.9	181	cT2N2M1	Yes, None	Chemo, Sorafenib, RT	Yes	11 months (dead)
2016	Motooka (32)	Male-69	Yes	Left segments 1 + 2	4.3	4497	cT2aN0M0	Yes, None	RS+ Chemo	Yes	51 months (alive)
2016	Qian (33)	Male-79	Yes	Right parahilar	2.7×2.6	NM	cT1cN0M0	NM	Erlotinib	Yes	NM
2016	Sun (34)	Male-59	Yes	Right upper lobe	4.5×3.8×3.5	Normal	pT2aN0M0	KI-67 20%	RS	Yes	23 months (alive)
2016	Wang (35)	Malr-56	Yes	Right upper lobe	4.0×4.1× 4.8	NM	cT2N1M0	NM	NM	NM	NM
2016	Grossman (4)	Male-54	Yes	Right upper lobe	5×4	Normal	M1 IV	Yes, None	Chemo	Yes	3 months (dead)
2017	Valle (36)	Male-61	NM	Left lung	NM	Normal	M1 IV	NM	①Chemo; ②RT	Yes	55months (dead)
2018	Li (37)	Male-52	Yes	Right upper lobe	NM	Normal	IVb	KI-67 60%; Yes, None;	Chemo	Yes	2 months (dead)
2018	Basse (38)	Male-43	Yes	Right lung		NM	IV	Yes, None; PD-L1-,dMMR;	①Chemo; ②Chemo; ③Durvalumab	Yes	NM
2019	Ayub (39)	Male-61	Yes	Right upper lobe	2.3	Normal	pT1bN0M0	NM	RS+RT	Yes	6 months (dead)
2019	Chen (40)	Male-53	No	Right upper lobe	5.3×3.5	3296	CT3N0M0	KI 67 20% Yes, EGFR T790M +	①RS+chemo; ②icotinib; ③osimertinib	Yes	29 months (alive)
2019	Kuan (41)	Male-47	Yes	Right upper lobe	14	Normal	cT4NxM0	Yes, None; PD-L1high-level staining.	Pulmonary lobectomy	Yes	4 months (dead)
2019	Li (7)	Male-71	No	Right lower lobe	7×4.5	79480	cT4N3M1	KI-67 80%; Yes, FAT +; *PD-L1-*, MSS;	Radio-Frequency Ablation+Anlotinib	Yes	4 months (dead)
2019	Shi (42)	Male-60	Yes	Right upper lobe	7.5×7.2	1210	pT3N0M0	KI-67 50%	RS+chemo	Yes	15 months (dead)
2019	Wang (43)	Male-70	Yes	Right upper lobe	6.0×4.6	Normal	IV	*Yes*, TP53 +	①Erlotinib; ②Chemo+bevacizumab	Yes	9 months (dead)
2019	Yang (44)	Male-70	Yes	Left lower lobe	6.4×5.5	Normal	pT3N1M0	Ki67 30%; Yes, None;	RS refused further chemotherapy	NO	18 months (dead)
2020	Chen (45)	Female-65	No	Bilateral lung	NM	6818	IV	Yes, K-RAS+; PD-L1≥1%, MSS;	①Chemo; ②Anlotinib; ③Sintilimab	Yes	53 months (dead)
2020	Chen (46)	Male-63	Yes	Left lung	NM	NM	IV	NM	Palliative surgery	Yes	4 months (dead)
2020	Tonyali (5)	Female-62	Yes	Left upper lobe	6	NM	cT4N1M0	Yes, None;	①Chemoradiation; ②Nivolumab	Yes	14 months (dead)
2020	Nargund (47)	Male-66	Yes	Left lower lobe	7.6×7.5×7.5	394	cT3N2M0	NM	Chemo	Yes	NM
		Male-65	Yes	Right upper lobe	6.5×4.5×6.6	993	cT2bN2M0	NM	Chemo	Yes	NM
2020	Wang (48)	Male-41	NM	Right upper lobe	5.2×5.8	Normal	cT3N3M0, IIIC	KI 67 60%; Yes, None;	RT	No	12 months (dead)
Current Case	Hou	Male-66	Yes	Right upper lobe	3.3×2.5×4.0	22323	cT2N2MO, IIIA	KI 67 60%; Yes, NRAS+, PIK3CA+; PD-L1-	①Chemoradiation; ②Anlotinib; ③Sorafenib+Sintilimab	Yes	13 months (dead)

RT, radiotherapy; Chemo, chemotherapy; RS, radical surgery; NM, not mentioned; ①, first-line therapy; ②, second-line therapy; ③, third-line therapy; None, the driver genes detected in the literature were wild-type.

### Case Presentation

**Figure 2:**
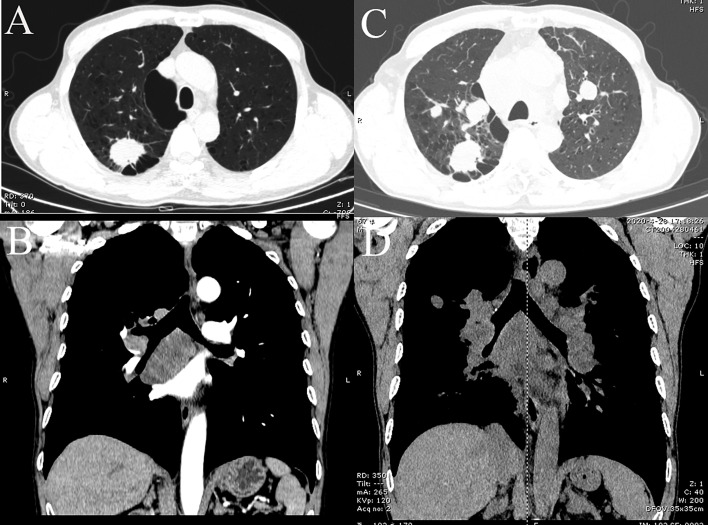
CT images of lung tumor and mediastinal lymph nodes of patient. **(A, B)** Baseline inspection on April 18, 2019, showed right upper lobe tumor and subcarinal lymph node metastasis, respectively. **(C, D)** Images on April 6, 2020, suggested multiple pulmonary metastases and multiple mediastinal lymph node metastases, respectively.

A 66-year-old Chinese male was admitted to hospital in April 2019 for cough and expectoration that had been accompanied by one incidence of hemoptysis. The enhanced CT scan (**Figures 2A, B**) identified a soft-tissue mass (3.3 × 2.5 × 4.0 cm) in the upper lobe of the right lung. The mass was considered to be lung cancer with subcarinal lymph node metastasis. However, no obvious abnormalities were found in the liver, stomach, and testes, and no tumor metastases were found on the enhanced MRI of the brain and centrum. A puncture biopsy of the lung tissue indicated non-small cell carcinoma and its morphology supported adenocarcinoma. Endobronchial ultrasound (E-BUS) lymph node examination was carried out in a different hospital and showed poorly differentiated carcinoma. Immunohistochemical analysis revealed that the mass was AFP (+) and hepatocyte (−). The patient’s liver function, viral hepatitis index, and immunological examination were normal, while the serum AFP value before treatment was 22,323 ng/ml (normal value: 0.0–8.0 ng/ml).

Next-generation sequencing (NGS) and programmed cell death-1 (PD-L1) testing in lung cancer tissues revealed that the EGFR, ALK, ROS-1, BRAF, MET, KRAS, HER2, AKT1, c-KIT, and RET genes were wild type, while the NRAS and PIK3CA genes were mutated. PD-L1 testing was negative.

**Figure 3:**
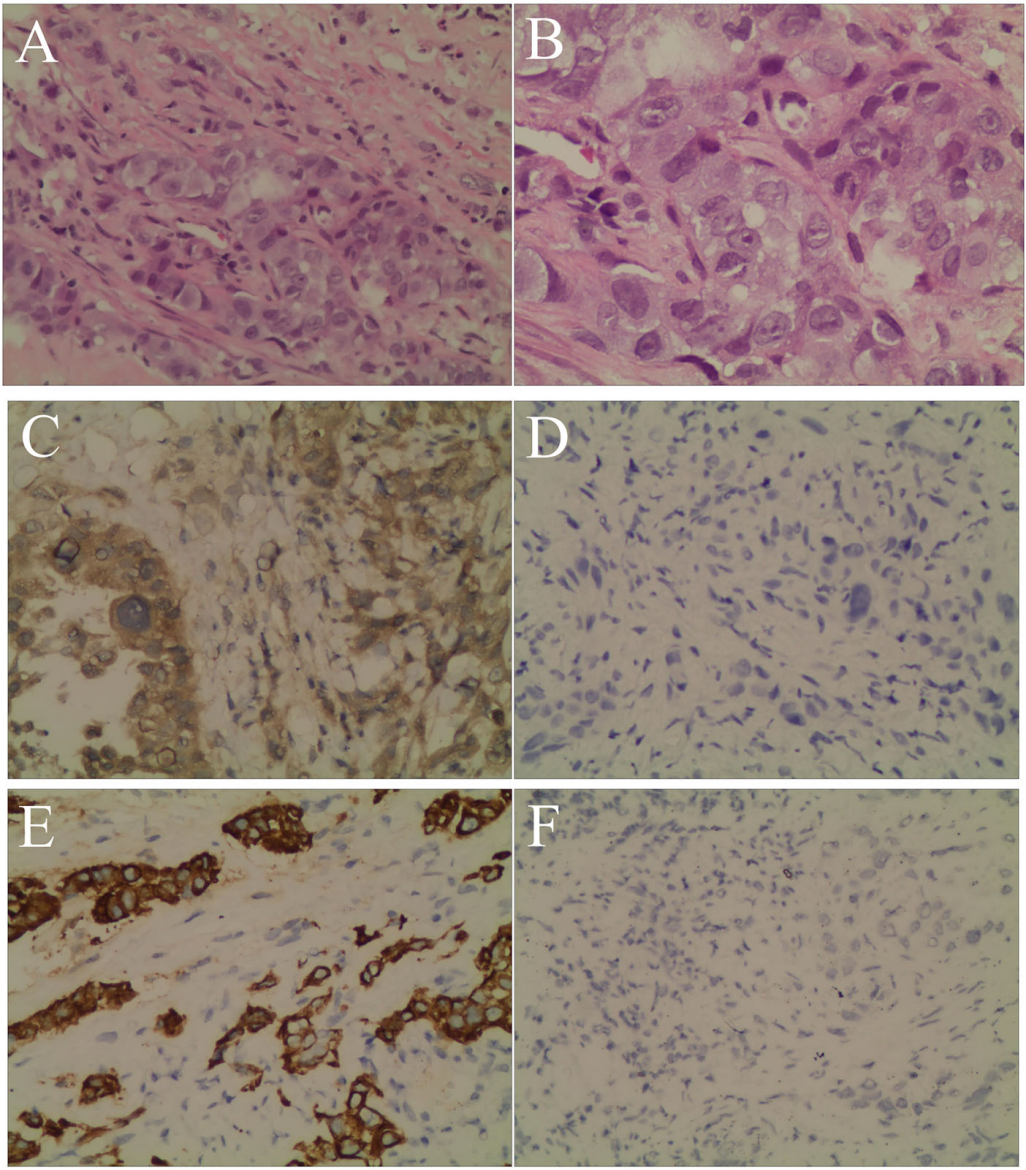
Hematoxylin and eosin staining and immunohistochemistry. **(A)** Neoplastic cells arranged in an alveolar pattern and the glandular lumen can be seen in the lesion area (magnification, ×200). **(B)** Neoplastic cells with prominent and hyperchromatic nucleoli, nuclear pleomorphism, and abundant eosinophilic cytoplasm, consistent with an adenocarcinoma with hepatoid differentiation (magnification, ×400). **(C)** The tumor cells were positive for GPC-3 (magnification, ×200). **(D)** The tumor cells were negative for hepatocyte (magnification, ×200). **(E)** The tumor cells were positive for CK7 (magnification, ×200). **(F)** The tumor cells were negative for TTF-1 (magnification, ×200).

The patient’s personal history indicated that the patient had a smoking index of 800, and denied any history of drinking. There was nothing special about his past medical history or family history. Combined with the above examinations, the patient’s diagnosis was adenocarcinoma of the right upper lobe cT2N2MO IIIA and he had no surgical indication. In May 2019, the patient received concurrent chemoradiotherapy (chemotherapy regimen: pemetrexed plus cisplatin), and the efficacy of the patient was evaluated as stable disease (SD). After two cycles of the original chemotherapy regimen, the patient’s efficacy was evaluated as progressive disease (PD). The patient later received two cycles of treatment with arotinib and his efficacy was also evaluated as PD. In April 2020, full-body enhanced CT examination revealed enlarged lung masses and increased mediastinal lymph nodes (**Figures 2C, D**), with intracranial and pyramidal metastases, and no obvious abnormalities in other sites. The serum AFP value was re-examined and found to be 10,075.99 ng/ml. A puncture biopsy of the cervical lymph node was performed and histological examination revealed an adenocarcinoma with hepatoid differentiation (**Figures 3A, B**). Immunohistochemical stains were positive for CK7, CK8/18, GPC-3, and ki67 (60%), and negative for arg-1, TTF-1, NapsinA, CD20, CDX2, P40, and PSA (**Figures 3C–F**). Thus, the diagnosis of stage cT4N3M1 HAL was made. The patient refused to undergo further genetic and PD-L1 tests. From April 10, 2020, the patient was treated with one cycle of sorafenib and sindilimab, and palliative radiotherapy was given to metastatic bone tumors. On April 28, 2020, the patient was admitted to hospital and his therapeutic effect was evaluated as PD by systemic re-examination. The patient’s condition worsened and he died in May 2020. The patient’s OS was 13 months after initial diagnosis.

### Case Features

The clinical characteristics of 51 cases are listed in **Table 2**. The average age of the patients was 59.9 ± 17.0 years old. There were 45 male cases, accounting for 88.2% of patients, and the percentage of smokers accounted for 87.2%. The average size of the primary tumor was 6.7 ± 1.5 cm. The tumors were located in the upper lobe of the right lung in 52.9% of cases. The proportion of serum AFP-positive patients before treatment, early-stage patients (TNM stage I and II), and patients who had received RS treatment were 69.2%, 34.1%, and 40%, respectively. Only a few HAL patients were reported as having a driver gene mutation and positive PD-L1.

**Table 2:** The clinical characteristics of 51 cases.

Characteristic		Result
Age (years)		59.9 ± 17.0
Gender		
Male		45
Female		6
Tumor location		
Right upper lobe		27
Not the right upper lobe		24
Tumor size(cm)		6.7 ± 1.5
Smoke		
Yes		34
No		5
Not mentioned		12
AFP		
Positive		27
Negative		12
Not mentioned		12
Stage at diagnosis		
I		6
II		8
III		11
IV		16
Not mentioned		10
Radical surgery		
Yes		20
No		30
Not mentioned		1
EGFR mutation		
Yes		1
No		14
Not mentioned		36
ALK mutation		
Yes		1
No		9
Not mentioned		41

### Survival Curve

**Figure 4:**
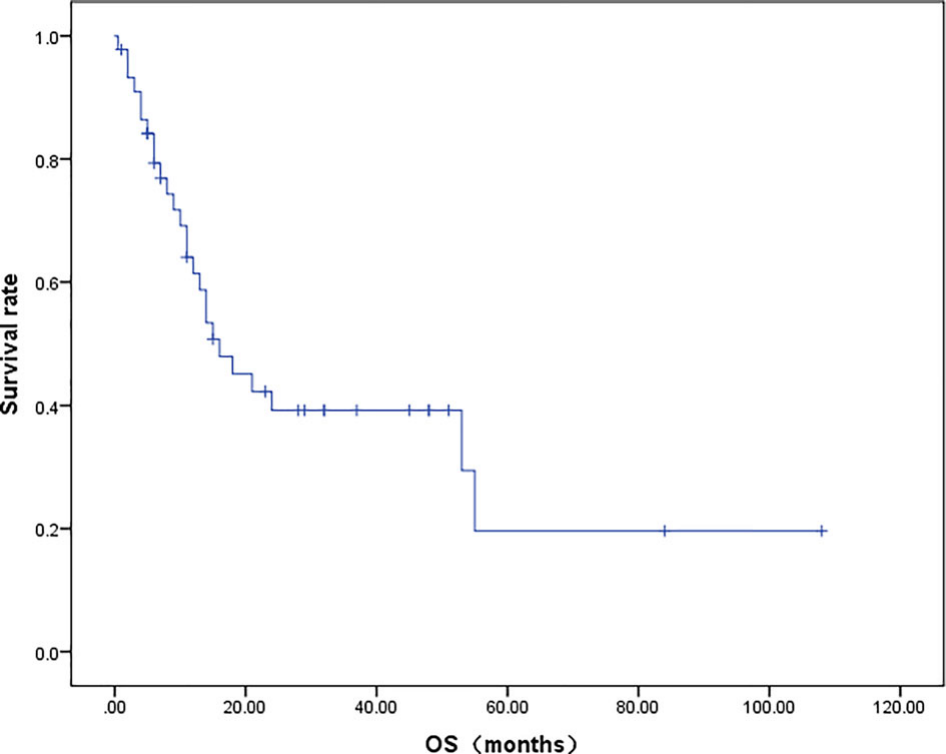
Survival curve of HAL patients.

A total of 42 patients were included in the OS analysis (**Figure 4**). The mOS of these patients was 16.0 months. The 2-year and 5-year survival rates of the patients were 35.3% and 8.0%, respectively. In the subgroup analysis, the 2-year survival rate for patients who received RS was 62.5%, while patients who did not undergo RS had a 2-year survival rate of only 12.5% (*p* = 0.009).

### Cox Proportional Hazards Regression Analysis

The Cox proportional hazards regression analysis was composed of 30 patients who had tumor size, location of tumor, serum AFP level before treatment, specific treatment methods, and survival data (**Table 3**). RS was shown to significantly improve the prognosis of HAL patients (HR 0.23; 95% CI: 0.075–0.71; *p* = 0.011). Compared with the right upper lobe group, there were no significant differences in the prognosis of the non-right upper lobe group (HR 0.89; 95% CI: 0.33–2.41; *p* = 0.82). The tumor size and serum AFP value before treatment also had no significant effect on the prognosis (HR 1.00, 95% CI: 0.86–1.20, *p* = 0.96; HR 1.00, 95% CI: 1.00–1.00, *p* = 0.25).

**Table 3:** Cox proportional hazard regression model.

	P	HR	95% CI	
Tumor location	0.82	0.89	0.33	2.41
Tumor size	0.96	1.00	0.86	1.16
Serum AFP	0.25	1.00	1.00	1.00
Radical surgery	0.011	0.23	0.08	0.71

## Discussion

Extrahepatic hepatoid adenocarcinoma can occur in gastric (63%), ovarian (10%), lung (5%), and uterine (4%) cancers (1). Okunaka et al. (11) defined HAL as lung adenocarcinoma with hepatoid differentiation or features of hepatocellular carcinoma and a positive serum AFP value. However, some studies have found that serum AFP levels can also be normal in HAL patients. Thus, the most recent definition of HAL is lung adenocarcinoma with hepatoid differentiation or features of hepatocellular carcinoma, with or without positive serum AFP values (34). The pathological H&E staining of our case shows large tumor cells, prominent and hyperchromatic nucleoli, nuclear pleomorphism, and abundant eosinophilic cytoplasm, consistent with adenocarcinomas with hepatoid differentiation. The cancer cells may be arranged in solid sheet nests or an alveolar pattern, and glandular lumen structures may be observed in the lesion area. Immunohistochemical analysis of HAL includes AFP, CK7, hepatocyte, GPC-3, and arg-1 staining, which may be partially or wholly positive. Primary HAL is mainly differentiated from primary pulmonary adenocarcinoma, lung metastases of hepatocellular carcinoma, lung metastases of hepatoid adenocarcinoma from other sites (such as the stomach, ovary, and uterus), and germ cell tumors, which can also cause an increase in AFP. Although the diagnosis of HAL relies on morphology, we hold that imagological examination and immunohistochemical analysis, including AFP, CK7, hepatocyte, GPC-3, TTF-1, arg-1, and CDX2 staining, are still important for the diagnosis and differential diagnosis of HAL based on our case study and other studies (48). The mechanism of AFP expression in this type of adenocarcinoma may be related to the homology of the lung and liver. Indeed, the lung and liver belong to primitive fore-gut derivatives during embryonic development, and abnormal differentiation of lung cancer cells tends to transform into hepatic cells, thus producing AFP (49).

This study highlights the fact that HAL is more common in middle-aged and elderly male smokers, and generally occurs in the right upper lobe of the lung. Among the patients, approximately 2/3 were serum AFP-positive before treatment. Once confirmed, approximately 2/3 of the patients were classified as middle and advanced stage (stage III and IV) according to the TNM staging system. The probability of EGFR mutation and ALK mutation in non-small cell lung cancer (NSCLC) patients is about 30% and 5%, respectively (50). In this systematic review, we found that the mutation rate of EGFR and ALK in all HAL patients who underwent genetic screening was 6.7% and 10.0%, respectively. Thus, we recommend that all confirmed HAL patients should undergo lung cancer-driven genetic screening, preferably NGS detection, PD-L1 testing, and microsatellite instability detection, to guide the gene and immunotherapy of HAL patients.

In our systematic review, we found that HAL patients had a poor prognosis, with a 5-year survival rate of only 8.0% and a 2-year survival rate of 35.3%. Due to the small number of HAL cases, there is no relevant guideline or expert consensus on the standard treatment method for this disease. We mainly refer to the National Comprehensive Cancer Network (NCCN) guidelines for lung adenocarcinoma for the treatment of HAL patients. In the current study, we found that HAL patients were mainly treated with RS, followed by chemotherapy with platinum-containing dual drugs and radiotherapy. Only a few patients underwent targeted therapy and immunotherapy.

We demonstrate for the first time that RS significantly prolongs the survival time of HAL patients. HAL patients undergoing chemoradiotherapy or chemotherapy had a 2-year survival rate of only 12.5%, indicating that patients who received this treatment were unable to achieve a long-term survival rate. In our case study, for example, the patient was not sensitive to both radiotherapy and chemotherapy, and achieved an OS of only 13 months. With respect to targeted therapy, Chen et al. (40) reported that a patient with EGFR T790M mutation receiving third-line therapy with osimertinib achieved progression-free survival (PFS) for 8 months. Khozin et al. (23) reported that the disease progression of a female patient with an ALK gene rearrangement was 6 months after treatment with crizotinib. Gavrancic et al. (31) reported a stage IV HAL case, who was treated with sorafenib combined with platinum-containing dual drugs, and achieved an OS of 11 months. In terms of immunotherapy, Basse et al. (38) reported a stage IV HAL patient with negative PD-L1 and mismatch genes repair defect (dMMR), who achieved partial response (PR) after third-line therapy with durvalumab. Here, we report disease progression in a patient with negative PD-L1 after third-line therapy with sorafenib and sintilimab. In contrast, another study described a patient with PD-L1 ≥1% who achieved PR after third-line therapy with docetaxel plus sintilimab (45). Taken together, these studies indicate that, even with combination therapy, HAL patients with PD-L 1 ≥1% or dMMR are likely to benefit from immunotherapy.

PIK3CA is a proto-oncogene, which can promote cell growth and induce the expression of anti-apoptotic genes after activation. The mutation rate of PIK3CA in NSCLC is about 2% (51). A PIK3CA inhibitor, alpelisib, is currently available and has been approved by the FDA for the treatment of advanced metastatic breast cancer with hormone receptor-positive, human EGFR2-negative, and PIK3CA mutation in men and postmenopausal women (52). Furthermore, its clinical phase II study in lung cancer is underway. In our case study, the efficacy of the three treatment schemes was poor, and thus, the use of a PIK3CA inhibitor may prove to be an effective treatment method.

Our study demonstrates for the first time that the serum AFP value before treatment is not related to the prognosis of HAL patients (HR 1.00, 95% CI: 1.00–1.00, *p* = 0.246). More studies are required to determine whether the change in serum AFP value before and after treatment has a significant impact on the prognosis of HAL patients. Some studies (53) have suggested that the pretreatment serum AFP heteroplasmon (AFP-L3) is an independent prognostic indicator of hepatic carcinoma. Serum AFP-L3 levels before treatment are negatively correlated with the prognosis of patients with hepatocellular carcinoma. Therefore, future studies could determine whether serum AFP-L3 can be used as an independent prognostic indicator for HAL patients by detecting pretreatment serum AFP-L3 levels.

In summary, HAL is a rare and special type of cancer that contains some features of both hepatocellular carcinoma and lung adenocarcinoma. HAL has similar cell morphology and intracellular antigens to hepatocellular carcinoma, while simultaneously containing some genetic mutations of pulmonary adenocarcinoma. The 5-year survival rate of HAL patients is low, and the 2-year survival rate of patients who do not receive RS is even lower. Thus, more research on gene-targeted and immunotherapy is required. The incidence of HAL is extremely low and less than 60 cases have been reported in the literature in the past 40 years. Therefore, the results of our study have certain limitations due to the small number of cases, and the epidemiology, molecular pathology, effective treatment regimens, and prognosis of HAL patients need to be further investigated.

## Data Availability Statement

The original contributions presented in the study are included in the article/supplementary material. Further inquiries can be directed to the corresponding author.

## Author Contributions

All authors listed have made a substantial, direct, and intellectual contribution to the work, and approved it for publication.

## Conflict of Interest

The authors declare that the research was conducted in the absence of any commercial or financial relationships that could be construed as a potential conflict of interest.

## Publisher’s Note

All claims expressed in this article are solely those of the authors and do not necessarily represent those of their affiliated organizations, or those of the publisher, the editors and the reviewers. Any product that may be evaluated in this article, or claim that may be made by its manufacturer, is not guaranteed or endorsed by the publisher.
